# Simulation-based detection of usability conflicts for an ISO/IEEE 11073 SDC workstation: a spine surgery case study

**DOI:** 10.1007/s11548-026-03753-6

**Published:** 2026-07-21

**Authors:** Okan Yilmaz, Simon Kißmann, Rastislav Pjontek, Linda Busch, Christian Blume, Klaus Radermacher, Armin Janß

**Affiliations:** 1 Chair of Medical Engineering, RWTH Aachen University, 52074 Aachen, Germany; 2 Department of Neurosurgery, University Hospital Aachen, 52074 Aachen, Germany

**Keywords:** ISO/IEEE 11073 SDC, Usability conflicts, Simulation framework, Spine surgery workflow, Medical device interoperability, Business process model and notation, BPMN

## Abstract

**Purpose:**

Open, vendor-independent networking of medical devices via ISO/IEEE 11073 Service-oriented Device Connectivity (SDC) enables central operating room (OR) workstations. Pure interoperability, however, does not guarantee usability or safety, especially when many devices, roles, and workflow phases interact in a dynamic and unpredictable manner [1]. We present a simulation framework that detects resource, workflow, and usability conflicts in SDC-based workstation setups, demonstrating its application to the procedure of dorsal decompression and spinal fusion.

**Methods:**

The framework combines three inputs: (1) a Business Process Model and Notation (BPMN) workflow of cervical decompression/fusion created together with surgeons, (2) device-specific User Interface (UI) Profiles that encode human–machine interaction requirements, and (3) a structured description of the available OR setup (screens, foot- and handswitches, roles, sterility zones). An assignment engine automatically allocates device functions to input/output resources and validates every assignment against a library of constraints. Detected violations (e.g., capacity, temporal relations, sterility zone, feedback conflicts) are reported, and a conflict-free configuration prototype cockpit interface is developed and evaluated by 11 clinicians.

**Results:**

Several types of conflict patterns could be detected through simulation, including input device shortages (pedal mismatches), output device limitations (inability to adequately display parameters), or role capacity overload (one person performs too many tasks). Adjusting the OR setup step by step resolved the existing usability violations, yielding a conflict-free assignment. A formative evaluation with 11 clinicians provided initial feedback indicating that the simulation-derived, conflict-free workstation layout is feasible and usable for the selected tasks in the studied procedure.

**Conclusion:**

The simulation cannot guarantee perfect usability or conflict-free operation. Its reliability ultimately depends on the fidelity of the BPMN workflow, the correctness and completeness of the encoded usability constraints, and the quality of the available UI Profiles. It nevertheless proves to be an efficient early-stage filter. Our results demonstrate that simulation-based conflict detection can reveal usability and safety issues in SDC OR setups prior to their installation.

## Introduction

The transition from proprietary, vendor-locked operating room systems to open, interoperable, and standardized solutions based on ISO/IEEE 11073 Service-oriented Device Connectivity (SDC) promises flexibility and scalability. The SDC standards facilitate interoperability between medical devices by providing a standardized nomenclature, service model, and communication protocol [2–4]. By eliminating the need to develop a communication protocol when collaborating, the workload could be reduced. Yet, pure semantic interoperability is not enough to guarantee that a multi-device setup is safe and usable.

In a typical OR setting, several clinicians (e.g., surgeon, assistant, OR nurse, surgical technologist, anesthesiologist) interact with heterogeneous devices via touchscreens, footswitches, or hardware knobs [5, 6]. The primary objectives of this study are to develop a simulation framework for detecting usability conflicts in SDC workstation setups and to evaluate its effectiveness through a case study involving spine surgery. Our work contributes to the field by providing a novel simulation-based approach that integrates BPMN process modeling with machine-readable UI requirements for detecting usability conflicts early in the development process.

When interoperable device functions are reassigned to a centralized SDC workstation and attached input/output elements, conflicts can arise [7]. For instance, sharing a configurable hand/footswitch could lead to situations where a device function cannot be used [8]. Limited device control capabilities (read-only) might mitigate risks, but they also reduce the capabilities of remote interventions [9]. Mandatory required information during a procedure (such as a microscope livestream) could be overlapped by warnings from other devices. Clinicians are also concerned that relying on automated suggestions might decrease their autonomy or critical thinking [10].

Verifying such issues traditionally relies on formative tests in early developmental stages, on summative evaluations, and expert reviews of risk management files [11, 12]. However, publishing those risk management documents poses a barrier for open interconnection of medical devices, as they contain sensitive information. To address those concerns, we introduced a machine-readable XML Schema, encoding UI Requirements, which are represented through human–machine interaction constraints [13]. In parallel, surgical workflow research has found that Business Process Model Notation (BPMN) can be used to model clinical processes with SDC device interactions [14, 15]. The present short communication uses these two concepts (BPMN-Process Model and machine-readable UI Requirements) to implement a simulation-based detection of usability conflicts. Alternatively, the process model could be represented using the Decision Model and Notation (DMN). However, DMN primarily targets explicit decision logic (e.g., decision tables) rather than workflow orchestration. Since our approach focuses on simulating defined tasks and checking machine-readable UI and OR constraints, we did not use DMN in this work.

The contribution of the work is threefold. A BPMN process model for the procedure of dorsal decompression and fusion of the cervical spine has been developed collaboratively with surgeons, incorporating detailed inputs regarding procedural steps, role responsibilities, and interaction requirements based on real-world surgical practices.

A simulation pipeline combined BPMN processes, UI Profiles (HMI characteristic for medical devices and their functions), and a structured OR setup description to detect resource, workflow, and usability conflicts. A first formative evaluation of the automatically derived, conflict-free workstation prototype was developed and evaluated.

## Materials and methods

### Architecture

**Fig. 1:**
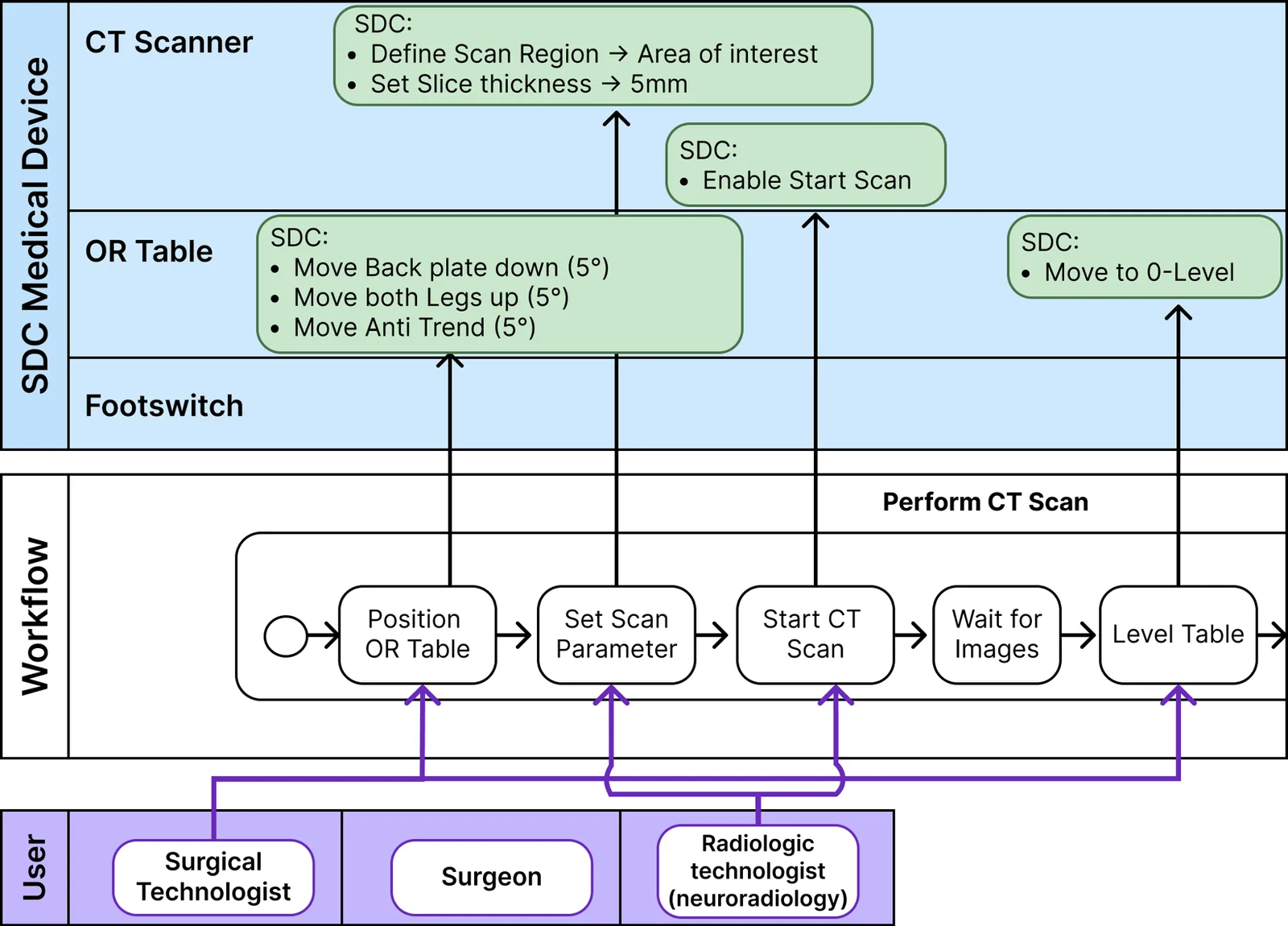
Excerpt from a BPMN process model for the surgical procedure: dorsal decompression and fusion of the cervical spine

The goal of the architecture is to combine the provided data into an executable model that can be automatically checked for violations (e.g., resource or input device conflicts) and iteratively optimized until a conflict-free SDC workstation configuration is found. The first input is the BPMN process model for the surgical procedure *dorsal decompression and fusion of the cervical spine*. Figure 1 shows a simplified excerpt of the procedure, with annotations for each workflow step in white, the responsible clinical role in purple, the involved device in light blue, and the concrete interaction with the medical device in green.

The OR setup is represented as a structured, machine-readable YAML file and serves as the second input to the framework. It is organized into categories for staff/roles, screens, input devices, and output devices. The role part includes the number of staff per role and whether a role operates in the sterile field. The screen model includes the attributes resolution, physical size/diagonal, DPI, maximum FPS, latency, sterility zone, and optional speaker properties. Input devices include the attributes input type (e.g., analog pedal), number of programmable functions, sterility zone, allowed roles, activation force, protection class, and control speed. Output devices include the attributes type (e.g., speaker, light indicator), max SPL, sterility zone, and (if applicable) haptic support. The last input is the UI Profile list, which includes machine-readable HMI requirements for each device [13]. An exemplary UI Profile for a high-frequency cutting device can be found in [16].

Overall, the study followed a two-step process. First, a YAML file representing an OR setup is loaded into the simulation and adjusted based on the simulation results. Second, a paper prototype GUI has been developed based on the final simulated (conflict-free) solution and has been evaluated by 11 participants.

### Assignment and constraint checking

All data is parsed into Python object models, and the BPMN processes are split. Tasks that occur at the same time share a step ID, and those that don’t get each a separate one. An in-house Python script performed an assignment, iterated over all steps, and attempted to map all input/output metrics to the corresponding input/output devices. The modality (pedal, touch), capacity (free pedals), zone compatibility (sterile, non-sterile), role permissions, and device-specific requirements have to be considered. For instance, sterility requirements are compared against role allocations to ensure that device interactions do not breach sterility, preventing contamination in the OR.

For each workflow step, the required device metrics must be mapped to available input/output resources. We first attempt a group assignment (mapping all interaction metrics to the same resource) to preserve coherence and minimize switches. If no single resource can host the complete set due to violated constraints (e.g., insufficient display capacity), we attempt to assign the metrics individually on the available resources. A simulation run iterates over all workflow steps sequentially. It can succeed once all steps are assigned without violating any constraints (Table 1**)**. It terminates early if, at any step, at least one required interaction metric cannot be mapped after trying grouped and metric-wise assignments across all eligible resources. The first non-executable step and the violated constraints are logged. The engine follows a deterministic first-fit strategy given a fixed ordering of steps/interactions and available resources. However, it does not guarantee optimal design/layout and, depending on ordering, may fail to find a feasible mapping even if one exists.

**Table 1:** Catalog of conflict types automatically checked by the simulation, with a short explanation for each type

Conflict type	Origin	Short description
Input capacity	Resources	The input device lacks enough programmable controls
Input type	Resources	No device offers the required modality (e.g., pedal, joystick, touch)
Input requirements	Resources	Device-specific specs (IP rating, force, speed, etc.) unmet
Zone compatibility	Sterility	Input device and assigned role lie in different sterility zones
Authorization	Policy	The role lacks permission/qualification to operate the function
Output capacity	Resources	Display surface/resolution is too small for the required information
Output type	Resources	No device supports the required output modality (e.g., image, audio)
Output requirements	Resources	Display specs (e.g., resolution, fps, latency, SPL, etc.) unmet
Feedback requirements	Feedback	Required visual/auditory/haptic feedback cannot be provided
Positioning	UI Presentation	Relative/absolute positions from the UI Profile cannot be kept
Visibility	UI Presentation	Always-visible data (pedal mapping, HF power, video) could be obscured
Workload	Workload	Role overloaded by parallel critical or too many simultaneous tasks

Future work should investigate optimization-based assignment approaches (e.g., formulating the mapping as a constrained optimization problem) to derive optimal or provably feasible configurations and to reduce the dependence of the result on the chosen ordering.

### Detected conflict types and constraints

The simulation framework identifies and categorizes various conflict types that can arise in SDC workstation setups. Those categories were collected after reviewing conflict types and an analysis of their applicability in interoperable solutions [11, 12, 17, 18]. Table 1 lists all conflict types and constraints that are checked during the simulation. It also contains an Origin column, which indicates the origin of the underlying requirement, so whether the constraint primarily comes from the **physical OR setup** (resources), **organizational rules** (policy), **sterility constraints** (sterility), **human factors considerations** (workload), or **UI-related requirements** (UI presentation and feedback).

### Formative usability evaluation—study design

The formative study aimed to gather early qualitative feedback on the conflict-free GUI workstation layout generated by the simulation and to identify any usability issues that had not been detected by the automated checks. It was not intended as a quantitative validation of the simulation’s conflict detection performance. Because we used paper-based static screens, the evaluation results focus on layout-related usability aspects (discoverability, readability, labeling consistency, and function-to-device mapping), not on interactive behavior. Interaction-based studies have already been performed for this type of OR Workstation [20].

#### Participants

The Department of Neurosurgery of the University Hospital Aachen participated with 2 surgeons, 2 nurses and 7 OR nurses/surgical technologists. Participants were familiar with the targeted procedure. The participants had an average of 9.55 ± 5.59 years of experience in their current roles.

#### Prototypes

We used a paper-based prototype: Three workstation screens were printed on paper and placed side by side on a table to emulate a multi-screen OR workstation. An SDC-compatible footswitch was placed in front of the participants, with its assignment displayed inside the central surgical cockpit screen.

#### Tasks

All participants worked through the same tasks (Table 2), which were extracted from the developed process model of dorsal cervical decompression and fusion.

**Table 2:** Tasks performed by the participants

Task	Description
1–7	Please point out the (HF device/surgical drill/OR table/OR light/footswitch/CT scanner/microscope stream)
8–11	Where is the (slide head/legs up/back up/emergency stop) function of the OR table?
12–14	What is the (rotation speed/flow/rotation direction) of the surgical drill?
15–16	What is the (intensity/diameter) of the OR light?
17–18	What is the (neutral electrode status/coagulation power for the connected instrument) of the HF device?
19–20	What happens when pressing the (blue/yellow) pedal of the footswitch?
21	What is the slice thickness of the CT scanner?
22	What is the microscope zoom level?

#### Procedure

After a brief standardized introduction to the paper-based workstation prototype, the moderator presented the study procedure and encouraged participants to verbalize their thoughts during task execution (think-aloud). The moderator then read each task aloud (Table 2), and the participant attempted to complete it using the printed multi-screen layout and a provided footswitch on the ground. One observer documented participants’ verbalized thoughts and interaction problems.

Task completion was logged as success (yes/no). If a participant did not reach a correct solution within 20 s, the moderator provided the solution, and the session continued with the next task. This predefined cutoff was chosen to keep the overall session duration comparable across participants and to avoid frustration in a static, non-interactive prototype setting. Giving the wrong answer resulted in immediate task failure. Afterward, participants completed a questionnaire based on Prümpers’ standardized ISO 9241-110 questionnaire, adapted for this task [19]; 13 Questions were answered using a 7-point Likert scale.

## Results

### Simulation output

Through test setups, all conflicts in Table 1 could be simulated and detected. We created an OR setup that we thought would suit the procedure of dorsal decompression and spinal fusion. This setup was run through the simulation framework, and several conflict types could be identified, including input device shortages, output device limitations, and role capacity overload. These conflicts were systematically addressed by adjusting the OR setup, such as adding additional displays or selecting alternative footswitches. Ultimately, a conflict-free configuration was achieved.

Three **monitors** were integrated into the setup. A touchscreen surgical cockpit (17.5″, 2540 × 1440) was used to monitor and control the OR table, OR light, drill, footswitch, and HF device. On a second screen, a microscope livestream (29″, 3840 × 2160) and, on a third, an intraoperative planning and navigation system are displayed (27″, 3840 × 2160). The font sizes and screen sizes were adjusted to ensure readability at the individual use distances. The presence and **sterility** requirements for each role (surgeons, nurses, circulators, assistants, and anesthesiologists) were meticulously defined to ensure proper workflow dynamics within the sterile and non-sterile fields. As **input devices**, a programmable footswitch with three pedals, a handswitch for the microscope, and a handswitch for the HF device were used. As an additional ** output device**, speakers ensured effective communication through the procedures and enabled auditory feedback.

### Formative evaluation

**Fig. 2:**
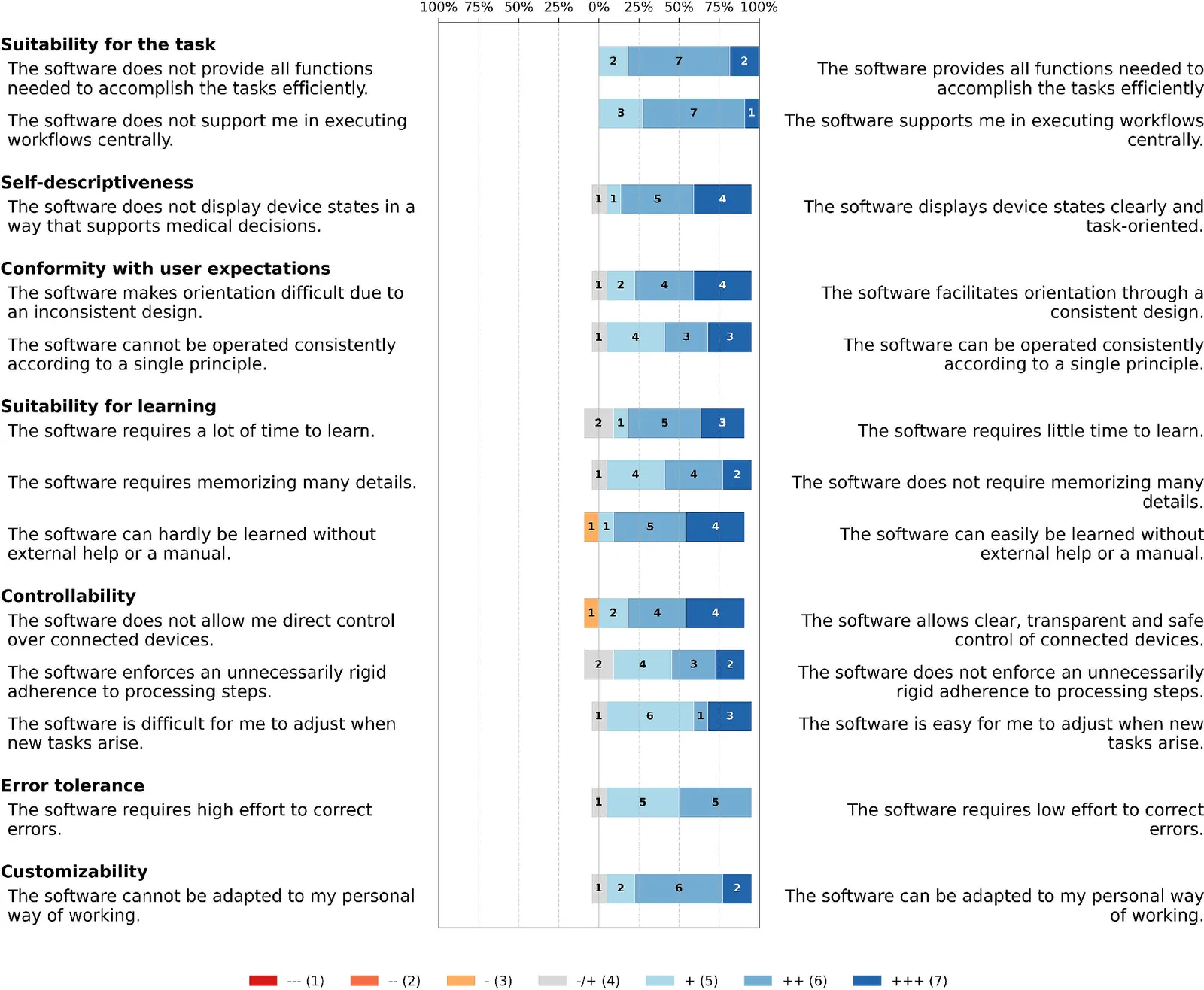
Horizontal diverging stacked bar charts of responses to 13 questionnaire statements, each rated on a 7-point Likert scale with a negative statement on the left and a positive statement on the right

The formative evaluation provided quantitative and qualitative insights into the usability and functionality of the simulation-wise conflict-free SDC workstation, not the simulation algorithm itself. Parts of the GUI concept have already been published elsewhere (e.g., [16, 20]), while other cockpit elements will be part of future publications. The answers to the questionnaires were generally positive, as shown in Fig. 2. The mean rating for every rated statement was closer to the right side (positive) than to the left side.

The success rate was 100% for all tasks except the following three: Task 10 (1 fail—“Where is the backup function of the OR table?”), Task 13 (1 fail—“What is the flow of the surgical drill?”), and Task 20 (2 fails—“What happens when pressing the yellow pedal of the Footswitch?”).

The thinking-aloud method provided additional valuable feedback.

- A consistent design helped support orientation
- Icons were seen as very important.
- Font sizes were rated as very good,
- Image of the table increased learnability.

However, specific areas were criticized:

- Displaying percentage values alone increased mental workload. Using a filled bar supports easier comprehension.
- Showing all features of the OR light was deemed excessive for this procedure.
- Showing the microscope light level is essential due to its direct impact on patient safety. High light levels can lead to rapid skin drying.
- Using the yellow pedal for a different task other than HF Cutting is unintuitive.
- Seeing an image of the operated table part while operating the OR table might increase learnability, but after learning the way it works, it takes up a lot of space.

### Insights compared to simulation output

The thinking-aloud method provided insights, which were mainly caused by (a) performance factors, (b) improper UI Profiles, and (c) situation-specific preferences.

- The user performance needs to be considered in more detail. Simply reviewing the criticality of a task or the number of tasks is not enough to estimate the stress it causes.
- UI Profiles for the microscope (and all other devices) should be developed together with device manufacturers, which did not happen for the microscope.
- Reducing OR light features can affect user experience during specific operations, but all functions must still be accessible during the procedure. A tradeoff could be made, where some properties are visible at all times, and others dynamically appear.

## Discussion & limitation

The integration of a simulation-based detection framework into the development phase of ISO/IEEE 11073 SDC workstations presents a promising advancement in reducing usability errors related to interoperability. By leveraging this model, developers can preemptively identify and resolve potential conflicts that arise from device interactions in complex operating room environments. This proactive approach not only enhances patient safety but also streamlines workflow efficiency, which is a benefit observed across integrated OR systems [21].

Our findings suggest that applying simulation frameworks during early-stage development allows for systematic identification of usability issues before they manifest in real-world settings. This capability is crucial given the dynamic nature of surgical procedures, where multiple devices and roles interact simultaneously, especially when used together with ISO/IEEE 11073 SDC. The ability to detect and mitigate input/output resource shortages, role capacity overloads, and device control limitations contributes significantly to creating safer and more efficient medical environments. This framework’s contribution lies in its focus on usability conflict detection in SDC standards, offering enhanced adaptability and scalability within open networked solutions.

Despite these promising results, several limitations still exist, which can be grouped into (i) simulation-related limitations and (ii) limitations identified through the formative study.

i. The simulation has so far been applied only to variations in a single surgical procedure. Applying the framework to a broader set of procedures is necessary to assess generalizability. Moreover, the current model detects conflicts only with respect to the predefined constraint catalog (Table 1). Extending the approach with models of user preferences could further improve the quality of the GUI layouts. Finally, this work did not empirically investigate the usability and safety implications of dynamic function reassignment across workstation components. Future studies should therefore evaluate interactive prototypes that deliberately vary function-to-device mappings during runtime and quantify effects on cognitive load and error probability.
ii. The formative evaluation was conducted using a paper-based prototype. Participants could not experience interaction dynamics. Accordingly, the findings primarily reflect layout, information, and mapping-related aspects. In addition, participant feedback indicates that expectations shaped by existing products and local practices should be considered. Lastly, the sample size was limited (n=11) and recruited from a single department, which restricts the external validity of the evaluation results.
